# Association between the PLTP rs4810479 SNP and Serum Lipid Traits in the Chinese Maonan and Han Populations

**DOI:** 10.1155/2021/9925272

**Published:** 2021-07-02

**Authors:** Fen-Han Zhang, Rui-Xing Yin, Li-Mei Yao, Wei-Xiong Lin, Jin-Zhen Wu, De-Zhai Yang

**Affiliations:** ^1^Department of Cardiology, Institute of Cardiovascular Diseases, The First Affiliated Hospital, Guangxi Medical University, Nanning, Guangxi, China; ^2^Department of Molecular Genetics, Medical Scientific Research Center, Guangxi Medical University, Nanning, Guangxi, China

## Abstract

The association between the phospholipid transfer protein (PLTP) gene rs4810479 single-nucleotide polymorphism (SNP) and serum lipid levels is largely unknown. This investigation aimed to evaluate the relationship between the PLTP rs4810479 SNP, several environmental risk factors, and serum lipid parameters in the Chinese Maonan and Han nationalities. Polymerase chain reaction-restriction fragment length polymorphism, gel electrophoresis, and direct sequencing were employed to determine the PLTP rs4810479 genotypes in 633 Maonan and 646 Han participants. The frequencies of CC, CT, and TT genotypes and the C allele were different between Maonan and Han groups (29.07%, 53.08%, 17.85%, and 55.61% vs. 35.60%, 49.70%, 14.70%, and 60.45%, respectively, *P* < 0.05). The C allele carriers in the Maonan group had higher high-density lipoprotein cholesterol levels than the C allele noncarriers, but this finding was only found in Maonan males but not in females. The C allele carriers in Han males had lower total cholesterol and low-density lipoprotein cholesterol levels than the C allele noncarriers. Serum lipid profiles were also affected by several traditional cardiovascular risk factors in both populations. There might be an ethnic- and/or sex-specific association between the PLTP rs4810479 SNP and serum lipid traits.

## 1. Introduction

Cardiovascular disease (CVD) is one of the leading causes of disability and early death worldwide, accounting for about one-third of the global mortality rate [1]. The cost of CVD constitutes a major economic burden to the society [2]. Many studies have proven that serum or plasma triglyceride (TG), low-density lipoprotein cholesterol (LDL-C), and high-density lipoprotein cholesterol (HDL-C) concentrations are independent risk factors for CVD [3–5].

It is well known that various genetic and environmental factors can lead to abnormalities of plasma lipids and lipoproteins [6–8]. Plasma lipid and lipoprotein concentrations are themselves highly heritable—estimates range from 40% to 60%. A number of genome-wide association studies (GWASes) have identified more than 95 genetic loci associated with plasma lipid phenotypes. One of the newly discovered loci is the phospholipid transfer protein (PLTP) gene [9–12].

PLTP (also called lipid transfer protein 2) is a member of lipid transfer/lipopolysaccharide- (LPS-) binding protein family. This family includes PLTP, LPS-binding protein (LBP), bactericidal/permeability-increasing protein (BPI), and cholesterol ester transfer protein (CETP) [13–15]. There are two molecular weights of PLTP, 55 kDa and 81 kDa. This may be due to different glycosylation [15]. PLTP is a monomeric and nonspecific lipid transfer protein, which can efficiently transfer free cholesterol, diacylglycerol, *α*-tocopherol, cerebroside, LPS, phospholipids, and sphingosine-1-phosphate [13–15]. There are two forms of lipoprotein-associated plasma PLTP (high active one and low active one) which are associated with apolipoprotein (Apo) A1- and ApoE-containing lipoproteins, respectively [16]. However, the cause for the existence of active and inactive PLTP in plasma is unclear. It is quite possible that PLTP might have other activities except its lipid transfer function [15]. PLTP is produced in various types of cells and secreted into plasma. It is highly expressed in human tissues such as the ovary, thymus, placenta, lung [17], liver, and small intestine and in macrophages [18, 19] and atherosclerotic lesions [19, 20]. The gene encoding PLTP (PLTP) is located on human chromosome 20. Its cDNA has a length of 1750 base pairs, including an open reading frame of 1518 nucleotides and a 3′-untranslated region (UTR) of 184 nucleotides. The mature PLTP contains 476 amino acids and 6 N-glycosylation sites that allow it to change its molecular weight (55 or 81 kDa) after different degrees of glycosylation modification [17]. Several previous studies have found that PLTP is an emerging cardiac metabolic factor which exerts a vital part in the development of blood lipid metabolism and atherosclerosis [21, 22]. PLTP is a main factor modulating the size and composition of high-density lipoprotein particles in the circulation and plays an important role in controlling plasma HDL-C levels [23]. PLTP deficiency in mice can lower total cholesterol (TC), HDL-C, and ApoA1 but increase TG levels significantly, impact the biological quality of high-density lipoprotein [24], and attenuate high-fat diet-induced insulin resistance and obesity [25]. Plasma PLTP activity (PLTPa) was significantly inversely correlated with carotid artery disease (CAAD), with a 9% decrease in odds of CAAD per 1 unit increase in PLTPa. Plasma TG levels, diabetes, statin use, and PLTP rs4810479 SNP were also associated with PLTPa significantly [26]. In human studies, both PLTP mass and PLTPa were associated with plasma lipid traits, glucose regulation, and atherosclerosis. Common variation at the PLTP structural locus region could explain about 30% of variation in PLTPa [27]. PLTP variants were associated with the PLTP mRNA level [28] and CVD risk [27, 29].

Being an isolated and conservative minority in China, the population of Maonan nationality was 107,166 (ranked 37) according to the statistics of China's sixth national census in 2010. They have own unique culture and life customs, such as intraethnic marriages, clothing, special lifestyle, and dietary structure. These characteristics are distinct from those in the largest ethnic group, Han Chinese. Therefore, we hypothesize that the genotype distribution and genetic traits of some lipid metabolism-related genes in the Maonan ethnic group may be different from those in the Han ethnic group. In the Chinese populations, there is no previous study to explore the association between the PLTP rs4810479 SNP and serum lipid levels. Thus, the aim of this study was to appraise the association between the PLTP rs4810479 SNP, several environmental risk factors, and serum lipid traits in the Maonan and Han nationalities.

## 2. Materials and Methods

### 2.1. Subjects

The study populations were stochastically chosen from our earlier stratified random specimens. The detailed inclusion and exclusion criteria have been described in a previous report [30]. In brief, all selected people were basically healthy and had no evidence of any chronic illness such as cardiac, hepatic, renal, or thyroid diseases. The participants who had a history of heart attack or myocardial infarction, stroke, congestive heart failure, and diabetes were excluded. They did not use medications known to affect serum lipid levels such as lipid-lowering drugs (statins or fibrates), *β*-blockers, diuretics, or hormones. The present study included 633 unrelated Maonan participants (251 males, 39.65%, and 382 females, 60.35%) and 646 unrelated Han subjects (268 males, 41.49%, and 378 females, 58.51%) [30]. The participants aged from 22 to 92 years (mean: 55.92 ± 14.30 years in Maonan and 54.50 ± 14.50 years in Han groups). The age structure and sex ratio between the two populations were matched. Basic information and health status of all participants refer to our previous study [31]. This research project was approved by the Ethics Committee of the First Affiliated Hospital, Guangxi Medical University (no. Lunshen-2014-KY-Guoji-001, March 7, 2014). All participants provided written informed consent before the study.

### 2.2. Epidemiological Survey

The survey was conducted using an internationally standardized method [32]. A standardized questionnaire was used to gather the information related to demographic statistics, socioeconomic status, and life style factors. Drinking and smoking were grouped according to daily consumption (0, ≤25, and >25 and 0, ≤20, and >20, respectively). Several parameters such as weight, body mass index (BMI), height, waist circumference, and blood pressure were also obtained.

### 2.3. Biochemical Measurements

After 12 hours of fasting, a cubital vein blood sample of 5 ml was obtained from all participants. Biochemical measurements including TC, TG, HDL-C, LDL-C, ApoA1, ApoB, and blood glucose were performed as previously described [33, 34].

### 2.4. DNA Amplification and Genotyping

Genomic DNA of the samples was extracted by the phenol-chloroform method [34]. Polymerase chain reaction-restriction fragment length polymorphism (PCR-RFLP) was utilized to determine the genotypes of the PLTP rs4810479 SNP. The forward and reverse primer pairs for PCR amplification were 5′-ATCCTCCGATCTTGGCTTCC-3′ and 5′-CCAGGTAGAGGGAACAGCAA-3′, respectively. The specific reaction condition was 5 min pretreatment at 95°C, denaturation at 95°C for 30 s, annealing at 59°C for 30 s, followed by extension for 40 s at 72°C for 33 cycles, and finally a 7 min extension at 72°C. The restriction enzyme was KpnI. After electrophoresis on 2.0% agarose gel containing 0.5 *μ*g/ml of ethidium bromide, the results were obtained under ultraviolet light. The PCR products of six samples were also confirmed by direct sequencing using ABI Prism 3100 (Applied Biosystems) in Shanghai Sangon Biological Engineering Technology & Services Co., Ltd., China.

### 2.5. Diagnostic Criteria

The normal reference values of serum lipid parameters including TG, TC, LDL-C, HDL-C, ApoA1, ApoB concentrations, and ApoA1/ApoB ratio as well as the diagnostic criteria of hyperlipidemia, hypertension, and type 2 diabetes, and normal weight, overweight, and obesity have been described in detail in our several previous research studies [30, 31, 33–35].

### 2.6. Statistical Analysis

The number of study samples in this research was estimated using Quanto software. All of the statistical analyses were accomplished using SPSS software (version 23.0). Normally distributed quantitative variables were expressed as mean ± standard deviation (nonnormally distributed serum TG levels were expressed as median and quartiles). Direct counting and standard goodness-of-fit test were used to determine the allele frequency and verify the Hardy–Weinberg equilibrium (HWE), respectively. The genotype distribution was tested by chi-square test, and the general features between the two ethnic groups were analyzed by unpaired *t*-test. The association between genotype and serum lipid parameters was assessed by the covariance analysis (ANCOVA), in which age, gender, blood pressure, BMI, cigarette smoking, and alcohol consumption were used as covariates. Stepwise modeling of multiple linear regression analyses was used to determine the relevant risk factors for serum lipid parameters in the Maonan, Han, male, and female (CC/CT genotypes = 1 and TT genotype = 2), respectively. Bilateral *P* value <0.05 was considered statistically significant.

## 3. Results

### 3.1. General Features and Serum Lipid Profiles

The features and serum lipid levels are presented in Table 1. The values of gender ratio, age structure, BMI, weight, and height; the percentages of cigarette smoking and alcohol intake; the levels of blood glucose, TC, LDL-C, ApoA1, and ApoB; and the ratio of ApoA1/ApoB were not different between the Maonan and Han populations (*P* > 0.05 for all). However, the levels of waist circumference, pulse pressure, diastolic and systolic blood pressures, and serum TG were higher, whereas the levels of HDL-C were lower in Maonan than in Han ethnic groups (*P* < 0.05 − 0.001).

### 3.2. Genotyping and Genotypes

**Figure 1:**
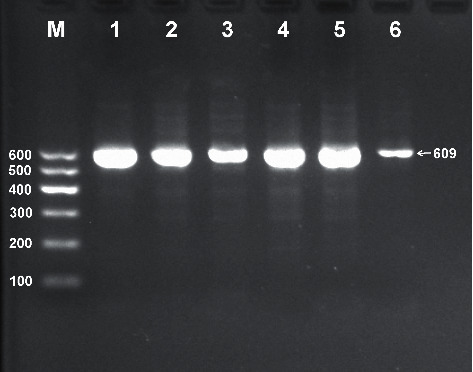
Electrophoresis of polymerase chain reaction products of the samples. Lane M is the 100–600 bp marker ladder; lanes 1–6 are samples; the 609 bp bands are the target genes.

**Figure 2:**
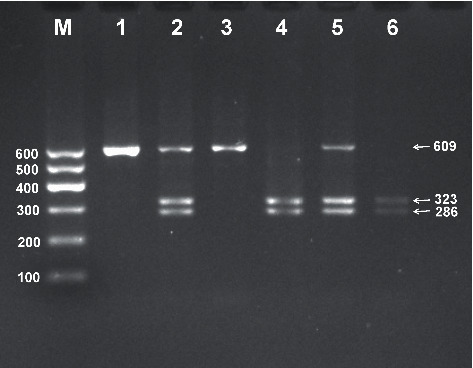
Genotyping of the PLTP rs4810479 SNP. Lane M, 100–600 bp marker ladder; lanes 1 and 3, TT genotype (609 bp); lanes 4 and 6, CC genotype (323 and 286 bp); lanes 2 and 5, CT genotype (609, 323, and 286 bp).

**Figure 3:**
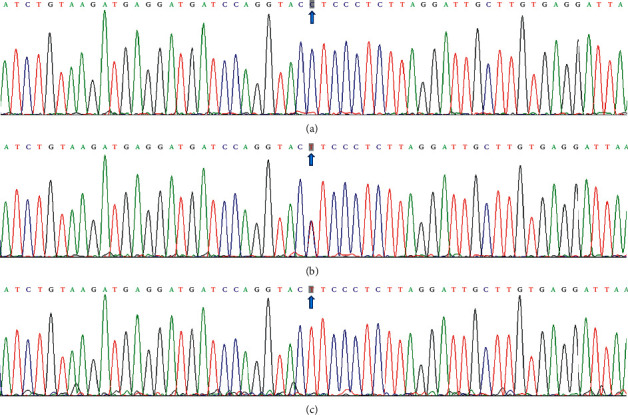
A part of the nucleotide sequence of the PLTP rs4810479 SNP: (a) CC genotype; (b) CT genotype; (c) TT genotype.

After electrophoresis of the PCR product, the products of 609 bp nucleotide sequences were observed in all samples (Figure 1). The bands of the three genotypes are presented in Figure 2: CT genotype (286, 323, and 609 bp), CC genotype (286 and 323 bp), and TT genotype (609 bp). The genotypes were distinguished by the presence of the enzyme restriction site (C allele) or absence (T allele). The results of direct sequencing of the samples are shown in Figure 3.

### 3.3. Genotype and Allele Frequencies

As shown in Table 2, there were significant differences in the frequencies of CC, CT, and TT genotypes and C allele between the Maonan and Han populations (29.07%, 53.08%, 17.85%, and 55.61% vs. 35.60%, 49.70%, 14.70%, and 60.45%, respectively, *P* < 0.05). However, the genotype and allele frequencies of the rs4810479 SNP in both ethnic groups were not significantly different between men and women (*P* > 0.05 for all).

### 3.4. Genotypes and Serum Lipid Concentrations

As summarized in Tables 3 and 4, serum HDL-C concentrations in the Maonan group were significantly different among the three genotypes (*P* < 0.05), and serum HDL-C concentrations were higher in the C allele carriers than the C allele noncarriers, but this finding was only restricted to males but not females. Lower TC and LDL-C concentrations in Han males were also observed in the C allele carriers than the C allele noncarriers (*P* < 0.05 for all).

### 3.5. Relevant Factors for Serum Lipid Parameters

Multiple linear regression analyses showed that serum HDL-C and ApoA1 concentrations in the Maonan group were correlated with the PLTP rs4810479 genotypes (*P* < 0.05; Table 5). Serum HDL-C concentrations in Maonan males, HDL-C and ApoA1 concentrations in Maonan females, and TC and LDL-C concentrations in Han males were associated with the genotypes (*P* < 0.05; Table 6). In addition to the PLTP rs4810479 genotypes, serum lipid traits in the participants were also influenced by several risk factors such as gender, age, waist circumference, BMI, pulse pressure, diastolic blood pressure, systolic blood pressure, fasting blood glucose, alcohol consumption, and cigarette smoking (*P* < 0.05 for all; Tables 5 and 6).

## 4. Discussion

The current study revealed that the Maonan ethnic group had higher TG and lower HDL-C concentrations than the Han ethnic group (*P* < 0.001 for each). There were no significant differences in the TC, LDL-C, ApoA1, and ApoB concentrations and the ApoA1/ApoB ratio between the Maonan and Han populations (*P* > 0.05 for all). It is common knowledge that dyslipidemia is one of the major changeable cardiovascular risk factors and is a major predictor of CVD mortality [1]. The difference in serum lipid profiles between the two populations may be due to distinct environmental, genetic factors and their interactions. Maonan nationality is one of 55 minorities in China. Being a mountain ethnic group, Maonan has its own unique history, custom, and culture, such as intraethnic marriages, specific clothing, inimitable lifestyle, and dietary habits. Most Maonan people are engaged in agricultural production, supplemented by animal husbandry, aquaculture, and other sideline industries. Rice and corn are their staple food, and pumpkin, sweet potato, and millet are the complementary foods. The preference for acidic food is the greatest feature of their diet culture. They have unique eating habits and lifestyles compared to other ethnic groups. Maonan ethnic group advocates intraethnic marriages. Their marriages are mostly arranged by parents. These results suggest that the genetic traits of some genes related to lipid metabolism may be different between the Maonan and Han ethnic groups.

According to the results of the International 1000 Genomes database (https://www.ncbi.nlm.nih.gov/variation/tools/1000genomes/), we knew that the rs4810479C allele frequency was 26.37% in British in England and Scotland (GBR); 27.27% in Utah residents (CEPH) with Northern and Western European Ancestry (CEU); 32.45% in Colombians from Medellin, Colombia (CLM); 33.84% in Finnish in Finland (FIN); 41.67% in African Caribbean individuals in Barbados (ACB); 42.62% in Americans of African Ancestry in the southwestern USA (ASW); 43.43% in Esan in Nigeria (ESN); 51.46% in Gujarati Indian from Houston, Texas (GIH); 55.34% in Han Chinese in Beijing, China (CHB); 57.56% in Bengali from Bangladesh (BEB); 64.29% in Southern Han Chinese (CHS); and 68.82% in Chinese Dai in Xishuangbanna, China (CDX). In the present study, we found that the Maonan ethnic group had lower rs4810479C allele frequency than the Han ethnic group (55.61% vs. 60.45%, *P* < 0.05). The genotype distribution of the PLTP rs4810479 SNP in the present study was also different between the two ethnic groups (*P* < 0.05), but there was no significant difference in the genotype and allele frequencies between males and females in both populations. These findings suggest that the PLTP rs4810479 SNP may have a racial/ethnic specificity.

The association between the PLTP rs4810479 SNP and serum lipid concentrations in different racial/ethnic groups is still largely unclear. In a previous GWAS, Musunuru et al. [36] prompted that the PLTP rs4810479 SNP was associated with HDL-C concentrations in European populations. In the present study, we noted that the PLTP rs4810479 SNP was significantly associated with several serum lipid phenotypes in the Maonan and Han populations. Subgroup analyses of serum lipid profiles according to sex showed that the C allele carriers had higher HDL-C concentrations in Maonan males and lower TC and LDL-C concentrations in Han males than the C allele noncarriers. These results indicate that there might be a race- and/or sex-specific association between the PLTP rs4810479 SNP and serum lipid traits in our study ethnic groups.

It is well known that serum lipid concentrations are also affected by many environmental risk factors such as population features, life style, diet structure, and physical inactivity [37]. In the current study, we also found that serum lipid concentrations were associated with several environmental risk factors in both ethnic groups. In a previous research study, we found that the intakes of total dietary fat, cholesterol, and energy were higher in Maonan than in Han ethnic groups [30]. The difference in living environment, eating habits, life style, and genetic background between the two populations may be the main cause of different serum lipid concentrations. Rice, corn, and other carbohydrate-rich foods are the daily staple foods of the Maonan people. They are also good at making various complementary foods with rice. They like to eat spicy and acidic foods that contain a lot of oil and salt. The intake of a large amount of carbohydrate, oil, and salt can increase the waist circumference and blood pressure in the Maonan people. Several studies have shown that long-term high-salt diet is an important risk factor to affect blood pressure levels [38, 39]. A meta-analysis showed that reduced sodium intake can lower blood pressure levels in people with or without hypertension [40]. In addition, the people of Maonan also like to eat pork, beef, and/or animal offals in a hot pot which is rich in saturated fatty acids. Many previous studies have shown that diet alone can explain the variation in blood lipid levels [41]. Long-term high-saturated fat diets are strongly associated with obesity, hypertension, dyslipidemia, and atherosclerosis [42, 43]. Therefore, different environmental risk factors such as unhealthy lifestyle and diet structure may further alter the association between genetic variation and blood lipid concentrations in our research populations.

Our work may have some limitations. First, we could not exclude the influence of diet and other environmental risk factors in the statistical analyses. Second, we also could not rule out the effect of asymptomatic diseases. Third, an association between the PLTP rs4810479 SNP and serum lipid concentrations was observed in this study, but many unmeasured factors should be considered including genetic and environmental risk factors. Finally, the sample size in our study populations is a bit small. Therefore, it is necessary to further expand the sample size, especially the gene-gene, gene-environment, and environment-environment interactions on serum lipid parameters to confirm our findings.

## 5. Conclusions

There was a significant difference in the genotype and allele distribution of the PLTP rs4810479 SNP between the Maonan and Han populations. The association between the PLTP rs4810479 SNP and serum lipid parameters was also different between the two nationalities and between males and females. There may be a racial/ethnic- and/or sex-specific association between the PLTP rs4810479 SNP and serum lipid concentrations in our study populations.

## Figures and Tables

**Table 1:** Comparison of demographic, lifestyle characteristics, and serum lipid levels between the Maonan and Han populations.

Parameter	Maonan	Han	*t* (*χ*^2^)	*P*
Number	633	646		
Male/female	251/382	268/378	0.446	0.531
Age (years)	55.92 ± 14.30	54.50 ± 14.50	1.761	0.079
Height (cm)	154.09 ± 8.22	154.88 ± 7.82	−1.756	0.079
Weight (kg)	53.58 ± 10.60	53.32 ± 8.88	0.459	0.646
Body mass index (kg/m^2^)	22.46 ± 3.62	22.20 ± 3.21	1.372	0.170
Waist circumference (cm)	76.73 ± 9.04	75.09 ± 8.05	3.415	0.001

Smoking status (*n* (%))				
Nonsmoker	500 (79.0)	484 (74.9)		
≤20 cigarettes/day	116 (18.3)	139 (21.5)	3.103	0.212
>20 cigarettes/day	17 (2.7)	23 (3.6)		

Alcohol consumption (*n* (%))				
Nondrinker	499 (78.8)	523 (81.0)		
≤25 g/day	71 (11.2)	59 (9.1)	1.547	0.461
>25 g/day	63 (10.0)	64 (9.9)		

Systolic blood pressure (mmHg)	134.49 ± 23.29	130.02 ± 19.73	3.697	<0.001
Diastolic blood pressure (mmHg)	83.29 ± 11.99	81.55 ± 11.01	2.698	0.007
Pulse pressure (mmHg)	51.20 ± 16.84	48.47 ± 15.77	2.989	0.003
Glucose (mmol/L)	6.08 ± 1.25	6.22 ± 1.33	−1.934	0.053
Total cholesterol (mmol/L)	4.99 ± 0.97	4.90 ± 0.93	1.640	0.101
Triglyceride (mmol/L)	1.28(0.88)	1.10(0.65)	4.750	<0.001
HDL-C (mmol/L)	1.62 ± 0.39	1.83 ± 0.42	−9.207	<0.001
LDL-C (mmol/L)	2.89 ± 0.81	2.84 ± 0.70	0.991	0.322
ApoA1 (g/L)	1.39 ± 0.23	1.38 ± 0.23	1.165	0.244
ApoB (g/L)	0.88 ± 0.19	0.87 ± 0.20	1.032	0.302
ApoA1/ApoB	1.66 ± 0.50	1.66 ± 0.45	0.013	0.990

HDL-C: high-density lipoprotein cholesterol; LDL-C: low-density lipoprotein cholesterol; Apo: apolipoprotein. The value of triglyceride was presented as median (interquartile range); the difference between the two ethnic groups was determined by the Wilcoxon–Mann–Whitney test.

**Table 2:** Comparison of the genotype and allele frequencies of the PLTP rs4810479 SNP in the Maonan and Han populations (*n* (%)).

Group	*n*	Genotype			Allele		*P*_HWE_
		CC	CT	TT	C	T	
Maonan	633	184 (29.07)	336 (53.08)	113 (17.85)	704 (55.61)	562 (44.39)	0.059
Han	646	230 (35.60)	321 (49.70)	95 (14.70)	781 (60.45)	511 (39.55)	0.319
*χ* ^2^			6.880		6.153		
*P*			0.032		0.014		

*Maonan*							
Male	251	78 (31.08)	128 (51.00)	45 (17.93)	284 (56.57)	218 (43.43)	0.549
Female	382	106 (27.79)	208 (54.29)	68 (17.92)	420 (54.97)	344 (45.03)	0.051
*χ* ^2^			0.919		0.314		
*P*			0.632		0.603		

*Han*							
Male	268	93 (34.70)	140 (52.24)	35 (13.06)	326 (60.82)	210 (39.18)	0.116
Female	378	137 (36.25)	181 (47.88)	60 (15.87)	455 (60.19)	301 (39.81)	0.987
*χ* ^2^			1.547		0.053		
*P*			0.461		0.862		

HWE: Hardy–Weinberg equilibrium. The genotype distribution between the two groups was analyzed by the chi-square test. The Hardy–Weinberg equilibrium was analyzed by the chi-square test of the goodness of fit.

**Table 3:** Comparison of the genotypes and serum lipid levels in the Maonan and Han populations.

Group/genotype	*n*	TC (mmol/L)	TG (mmol/L)	HDL-C (mmol/L)	LDL-C (mmol/L)	ApoA1 (g/L)	ApoB (g/L)	ApoA1/ApoB
Maonan	633							

CC	184	5.00 ± 0.96	1.22 (0.90)	1.61 ± 0.39	2.91 ± 0.83	1.39 ± 0.23	0.89 ± 0.19	1.64 ± 0.55
CT	336	5.02 ± 0.97	1.29 (0.87)	1.65 ± 0.40	2.90 ± 0.79	1.41 ± 0.24	0.88 ± 0.19	1.68 ± 0.49
TT	113	4.89 ± 0.96	1.35 (0.86)	1.53 ± 0.34	2.79 ± 0.81	1.34 ± 0.22	0.86 ± 0.19	1.62 ± 0.42
*F*		0.779	2.052	3.281	1.366	2.756	1.173	0.329
*P*		0.459	0.358	0.038	0.256	0.064	0.310	0.720
CC/CT	520	5.01 ± 0.97	1.26 (0.88)	1.64 ± 0.40	2.91 ± 0.81	1.40 ± 0.24	0.89 ± 0.19	1.67 ± 0.51
*F*		1.509	−1.389	4.844	2.712	3.721	2.109	0.024
*P*		0.220	0.165	0.028	0.100	0.054	0.147	0.876

Han	646							
CC	230	4.83 ± 0.88	1.11 (0.47)	1.88 ± 0.44	2.78 ± 0.59	1.39 ± 0.22	0.87 ± 0.20	1.67 ± 0.44
CT	321	4.94 ± 0.92	1.12 (0.74)	1.79 ± 0.41	2.85 ± 0.73	1.36 ± 0.23	0.87 ± 0.19	1.65 ± 0.46
TT	95	4.98 ± 1.04	0.98 (0.87)	1.85 ± 0.41	2.97 ± 0.83	1.38 ± 0.23	0.88 ± 0.21	1.65 ± 0.50
*F*		1.409	3.369	2.719	2.710	1.059	0.394	0.070
*P*		0.245	0.186	0.067	0.067	0.347	0.674	0.933
CC/CT	551	4.89 ± 0.91	1.11 (0.62)	1.82 ± 0.42	2.82 ± 0.68	1.38 ± 0.23	0.87 ± 0.20	1.66 ± 0.45
*F*		0.774	−1.578	1.132	3.615	0.457	0.493	0.034
*P*		0.379	0.144	0.288	0.058	0.499	0.483	0.853

TC: total cholesterol; TG: triglyceride; HDL-C: high-density lipoprotein cholesterol; LDL-C: low-density lipoprotein cholesterol; ApoA1: apolipoprotein A1; ApoB: apolipoprotein B; ApoA1/ApoB: the ratio of apolipoprotein A1 to apolipoprotein B. The value of TG was presented as median (interquartile range); the difference between the genotypes was determined by the Wilcoxon–Mann–Whitney test.

**Table 4:** Comparison of the genotypes and serum lipid levels between males and females in the Maonan and Han populations.

Ethnic/genotype	*n*	TC (mmol/L)	TG (mmol/L)	HDL-C (mmol/L)	LDL-C (mmol/L)	ApoA1 (g/L)	ApoB (g/L)	ApoA1/ApoB
Maonan/male								

CC/CT	206	4.90 ± 0.89	1.33 (0.98)	1.59 ± 0.40	2.82 ± 0.79	1.39 ± 0.27	0.88 ± 0.18	1.67 ± 0.60
TT	45	4.90 ± 0.94	1.53 (1.52)	1.43 ± 0.30	2.74 ± 0.81	1.33 ± 0.21	0.89 ± 0.18	1.55 ± 0.39
*F*		0.002	−1.350	4.244	0.860	0.718	0.106	0.608
*P*		0.962	0.177	0.040	0.355	0.398	0.745	0.436

Maonan/female								
CC/CT	314	5.09 ± 1.01	1.21 (0.82)	1.67 ± 0.40	2.96 ± 0.81	1.41 ± 0.21	0.89 ± 0.19	1.66 ± 0.46
TT	68	4.88 ± 0.99	1.21 (0.61)	1.59 ± 0.36	2.83 ± 0.81	1.35 ± 0.22	0.84 ± 0.19	1.67 ± 0.43
*F*		1.799	−0.800	1.639	1.302	3.341	3.365	0.135
*P*		0.181	0.424	0.201	0.255	0.068	0.067	0.714

Han/male								
CC/CT	233	4.95 ± 0.86	1.16 (0.78)	1.78 ± 0.44	2.85 ± 0.63	1.37 ± 0.26	0.92 ± 0.21	1.58 ± 0.48
TT	35	5.31 ± 1.06	1.09 (0.61)	1.84 ± 0.43	3.22 ± 0.87	1.41 ± 0.27	0.98 ± 0.22	1.52 ± 0.53
*F*		5.189	−0.974	0.835	9.517	1.250	2.797	0.179
*P*		0.024	0.330	0.362	0.002	0.264	0.096	0.672

Han/female								
CC/CT	318	4.84 ± 0.93	1.07 (0.54)	1.86 ± 0.41	2.80 ± 0.71	1.38 ± 0.21	0.83 ± 0.18	1.72 ± 0.41
TT	60	4.79 ± 0.98	0.96 (0.93)	1.86 ± 0.40	2.83 ± 0.77	1.36 ± 0.21	0.83 ± 0.18	1.73 ± 0.46
*F*		0.046	−1.143	0.396	0.167	0.005	0.128	0.262
*P*		0.831	0.253	0.529	0.683	0.946	0.721	0.609

TC: total cholesterol; TG: triglyceride; HDL-C: high-density lipoprotein cholesterol; LDL-C: low-density lipoprotein cholesterol; ApoA1: apolipoprotein A1; ApoB: apolipoprotein B; ApoA1/ApoB: the ratio of apolipoprotein A1 to apolipoprotein B. The value of triglyceride was presented as median (interquartile range); the difference among the genotypes was determined by the Wilcoxon–Mann–Whitney test.

**Table 5:** Relationship between serum lipid parameters and relative factors in the Maonan and Han populations.

Lipid	Risk factor	B	Std. error	Beta	*t*	*P*
*Maonan and Han*						

TC	Waist circumference	0.018	0.003	0.162	5.487	<0.001
	Age	0.007	0.002	0.106	3.688	<0.001
	Height	−0.010	0.003	−0.088	−2.987	0.003
	Diastolic blood pressure	0.005	0.002	0.066	2.293	0.022

TG	Waist circumference	0.038	0.003	0.302	11.155	<0.001
	Alcohol consumption	0.298	0.073	0.109	4.106	<0.001
	Glucose	0.069	0.022	0.082	3.142	0.002
	Diastolic blood pressure	0.008	0.003	0.081	3.024	0.003

HDL-C	Waist circumference	−0.012	0.002	−0.245	−6.804	<0.001
	Ethnic group	0.189	0.022	0.225	8.779	<0.001
	Alcohol consumption	0.135	0.033	0.128	4.045.	<0.001
	Gender	0.123	0.031	0.143	3.996	<0.001
	Age	0.003	0.001	0.090	3.163	0.002
	Body mass index	−0.010	0.004	−0.082	−2.338	0.020
	Pulse pressure	−0.002	0.001	−0.060	−2.123	0.034
	Cigarette smoking	0.067	0.033	0.067	1.987	0,047

LDL-C	Waist circumference	0.017	0.003	0.199	6.917	<0.001
	Age	0.007	0.001	0.125	4.499	<0.001
	Height	−0.009	0.003	−0.096	−3.291	0.001

ApoA1	Waist circumference	−0.003	0.001	−0.103	−2.453	0.014
	Alcohol consumption	0.152	0.019	0.264	7.792	<0.001
	Gender	0.076	0.018	0.162	4.191	<0.001
	Cigarette smoking	0.064	0.019	0.117	3.311	0.001
	Weight	−0.003	0.001	−0.127	−2.831	0.005

ApoB	Waist circumference	0.006	0.001	0.280	10.346	<0.001
	Age	0.002	0.000	0.136	4.914	<0.001
	Diastolic blood pressure	0.001	0.000	0.073	2.624	0.009
	Glucose	0.010	0.004	0.065	2.399	0.017

ApoA1/ApoB	Waist circumference	−0.014	0.002	−0.244	−6.668	<0.001
	Alcohol consumption	0.214	0.038	0.180	5.552	<0.001
	Gender	0.161	0.035	0.166	4.532	<0.001
	Age	−0.003	0.001	−0.082	−3.065	0.002
	Body mass index	−0.015	0.005	−0.107	−2.998	0.003
	Cigarette smoking	0.089	0.039	0.079	2.294	0.022
	Glucose	−0.022	0.010	−0.060	−2.234	0.026

*Maonan*						
TC	Waist circumference	0.025	0.004	0.233	5.810	<0.001
	Age	0.011	0.003	0.165	4.291	<0.001
	Gender	0.285	0.079	0.144	3.601	<0.001

TG	Waist circumference	0.038	0.004	0.345	9.209	<0.001
	Glucose	0.084	0.029	0.106	2.904	0.004
	Diastolic blood pressure	0.007	0.003	0.089	2.367	0.018
	Alcohol consumption	0.201	0.090	0.083	2.227	0.026

HDL-C	Waist circumference	−0.014	0.002	−0.333	−8.635	<0.001
	Genotype	−0.112	0.038	−0.110	−2.971	0.003
	Alcohol consumption	0.150	0.044	0.157	3.444	0.001
	Gender	0.094	0.038	0.117	2.503	0.013

LDL-C	Waist circumference	0.023	0.004	0.262	6.566	<0.001
	Gender	0263	0.066	0.160	4.018	<0.001
	Age	0.009	0.002	0.152	3.981	<0.001

ApoA1	Waist circumference	−0.006	0.001	−0.219	−5.605	<0.001
	Alcohol consumption	0.140	0.027	0.245	5.132	<0.001
	Gender	0.101	0.026	0.211	3.909	<0.001
	Cigarette smoking	0.082	0.029	0.143	2.829	0.005
	Genotype	−0.054	0.023	−0.089	−2.361	0.019

ApoB	Waist circumference	0.008	0.001	0.385	10.056	<0.001
	Age	0.002	0.000	0.181	4.945	<0.001
	Gender	0.042	0.015	0.109	2.866	0.040

ApoA1/ApoB	Waist circumference	−0.022	0.002	−0.390	−10.608	<0.001
	Alcohol consumption	0.224	0.045	0.184	4.499	<0.001
	Age	−0.004	0.001	−0.110	−3.023	0.003

*Han*						
TC	Diastolic blood pressure	0.011	0.003	0.128	3.153	0.002
	Age	0.006	0.003	0.098	2.478	0.013
	Body mass index	0.024	0.012	0.085	2.126	0.034

TG	Waist circumference	0.039	0.006	0.266	6.837	<0.001
	Alcohol consumption	0.370	0.114	0.123	3.249	0.001
	Diastolic blood pressure	0.008	0.004	0.077	1.995	0.046

HDL-C	Waist circumference	−0.011	0.003	−0.215	−3.871	<0.001
	Body mass index	−0.016	0.007	−0.120	−2.159	0.031

LDL-C	Age	0.007	0.002	0.155	4.003	<0.001
	Body mass index	0.031	0.008	0.140	3.608	<0.001

ApoA1	Alcohol consumption	0.168	0.023	0.288	7.189	<0.001
	Weight	−0.007	0.001	−0.282	−7.052	<0.001

ApoB	Waist circumference	0.005	0.001	0.189	4.863	<0.001
	Gender	−0.100	0.018	−0.248	−5.554	<0.001
	Diastolic blood pressure	0.003	0.001	0.142	3.766	<0.001
	Glucose	0.018	0.005	0.121	3.286	0.001
	Height	−0.003	0.001	−0.121	−2.675	0.008

ApoA1/ApoB	Body mass index	−0.039	0.005	−0.275	−7.287	<0.001
	Glucose	−0.042	0.013	−0.124	−3.360	<0.001
	Gender	0.213	0.040	0.232	5.359	0.001
	Alcohol consumption	0.231	0.051	0.200	4.540	<0.001
	Diastolic blood pressure	−0.004	0.002	−0.105	−2.824	0.005

TC: total cholesterol; TG: triglyceride; HDL-C: high-density lipoprotein cholesterol; LDL-C: low-density lipoprotein cholesterol; ApoA1: apolipoprotein A1; ApoB: apolipoprotein B; ApoA1/ApoB: the ratio of apolipoprotein A1 to apolipoprotein B; B: unstandardized coefficient; Beta: standardized coefficient.

**Table 6:** Relationship between serum lipid parameters and relative factors in the males and females of the Han and Maonan populations.

Lipid	Risk factor	B	Std. error	Beta	*t*	*P*
*Maonan/male*						

TC	Weight	0.029	0.005	0.332	5.320	<0.001
	Glucose	0.103	0.041	0.151	2.527	0.012
	Pulse pressure	−0.010	0.003	−0.190	−2.831	0.005
	Age	0.010	0.004	0.161	2.276	0.024

TG	Waist circumference	0.054	0.008	0.401	6.975	<0.001
	Glucose	0.179	0.054	0.191	3.321	0.001
	Alcohol consumption	0.365	0.142	0.148	2.574	0.011

HDL-C	Waist circumference	−0.015	0.002	−0.353	−6.195	<0.001
	Alcohol consumption	0.170	0.044	0.221	3.883	<0.001
	Genotype	−0.134	0.057	−0.133	−2.348	0.020

LDL-C	Weight	0.024	0.005	0.310	4.954	<0.001
	Alcohol consumption	−0.203	0.095	−0.128	−2.123	0.035
	Pulse pressure	−0.010	0.003	−0.212	−3.143	0.002
	Age	0.009	0.004	0.164	2.297	0.022

ApoA1	Alcohol consumption	0.153	0.031	0.293	4.898	<0.001
	Waist circumference	−0.005	0.002	−0.186	−3.231	0.001
	Cigarette smoking	0.085	0.031	0.162	2.713	0.007

ApoB	Waist circumference	0.005	0.002	0.234	2.342	0.020
	Glucose	0.025	0.008	0.183	3.254	0.001
	Pulse pressure	−0.002	0.001	−0.200	−3.164	0.002
	Age	0.002	0.001	0.179	2.638	0.009
	Weight	0.004	0.002	0,242	2.319	0.021

ApoA1/ApoB	Waist circumference	−0.021	0.004	−0.343	−6.021	<0.001
	Alcohol consumption	0.301	0.064	0.267	4.691	<0.001
	Systolic blood pressure	0.003	0.001	0.131	2.313	0.022

*Maonan/female*						
TC	Age	0.019	0.004	0.271	5.469	<0.001
	Waist circumference	0.024	0.006	0.197	3.997	<0.001
	Glucose	−0.085	0.042	−0.102	−2.035	0.043

TG	Waist circumference	0.033	0.004	0.357	7.518	<0.001
	Age	0.008	0.003	0.143	3.009	0.003

HDL-C	Waist circumference	−0.014	0.002	−0.288	−5.861	<0.001
	Genotype	−0.100	0.050	−0.098	−1.992	0.047

LDL-C	Age	0.013	0.003	0.235	4.870	<0.001
	Waist circumference	0.020	0.005	0.209	4.264	<0.001
	Alcohol consumption	0.619	0.297	0.102	2.086	0.038

AopA1	Waist circumference	−0.006	0.001	−0.221	−4.423	<0.001
	Genotype	−0.067	0.028	−0.120	−2.406	0.017

ApoB	Waist circumference	0.008	0.001	0.351	7.481	<0.001
	Age	0.004	0.001	0.276	5.847	<0.001
	Glucose	−0.016	0.008	−0.097	−2.027	0.043

ApoA1/ApoB	Waist circumference	−0.020	0.003	−0.377	−7.697	<0.001
	Systolic blood pressure	−0.003	0.001	−0.168	−3.545	<0.001
	Height	0.010	0.004	0.139	2.907	0.004

*Han/male*						
TC	Diastolic blood pressure	0.014	0.005	0.187	3.121	0.005
	Genotype	0.360	0.159	0.136	2.269	0.024

TG	Waist circumference	0.051	0.010	0.290	4.939	<0.001
	Alcohol consumption	0.380	0.165	0.135	2.302	0.022

HDL-C	Weight	−0.016	0.003	−0.299	−4.959	<0.001
	Alcohol consumption	0.133	0.053	0.151	2.504	0.013

LDL-C	Genotype	0.372	0.119	0.186	3.136	0.002
	Body mass index	0.029	0.012	0.149	2.507	0.013
	Glucose	0.060	0.027	0.132	2.230	0.027

ApoA1	Alcohol consumption	0.193	0.030	0.368	6.383	<0.001
	Weight	−0.008	0.002	−0.276	−4.784	<0.001

ApoB	Body mass index	0.012	0.004	0.197	3.322	0.001
	Glucose	0.028	0.008	0.199	3.426	0.001
	Diastolic blood pressure	0.003	0.001	0.159	2.681	0.008

ApoA1/ApoB	Weight	−0.019	0.003	−0.337	−5.821	<0.001
	Alcohol consumption	0.234	0.058	0.239	4.050	<0.001
	Glucose	−0.051	0.019	−0.156	−2.717	0.007

*Han/female*						
TC	Age	0.014	0.004	0.208	3.986	<0.001
	Height	−0.020	0.008	−0.130	−2.505	0.013

TG	Waist circumference	0.029	0.006	0.234	4.573	<0.001
	Diastolic blood pressure	0.014	0.005	0.145	2.825	0.005

HDL-C	Waist circumference	−0.009	0.004	−0.184	−2.259	0.024
	Body mass index	−0.023	0.011	−0.169	−2.077	0.039

LDL-C	Age	0.014	0.003	0.265	5.363	<0.001
	Body mass index	0.027	0.012	0.112	2.275	0.023

ApoA1	Body mass index	−0.015	0.003	−0.220	−4.364	<0.001
ApoB	Age	0.004	0.001	0.286	5.829	<0.001
	Body mass index	0.012	0.003	0.205	4.241	<0.001
	Cigarette smoking	−0.156	0.058	−0.130	−2.663	0.008

ApoA1/ApoB	Age	−0.006	0.001	−0.209	−4.279	<0.001
	Cigarette smoking	0.413	0.133	−0.151	3.096	0.002
	Body mass index	−0.040	0.007	−0.287	−5.970	<0.001

TC: total cholesterol; TG: triglyceride; HDL-C: high-density lipoprotein cholesterol; LDL-C: low-density lipoprotein cholesterol; ApoA1: apolipoprotein A1; ApoB: apolipoprotein B; ApoA1/ApoB: the ratio of apolipoprotein A1 to apolipoprotein B; B: unstandardized coefficient; Beta: standardized coefficient. The correlation among serum lipid parameters and the genotypes and several environmental factors was determined by multivariable linear regression analyses with stepwise modeling.

## Data Availability

The datasets generated during the present study are not publicly available because detailed genetic information of each participant was included in these materials.

## Abbreviations

ANCOVA: Analysis of covariance
Apo: Apolipoprotein
BMI: Body mass index
BPI: Bactericidal/permeability-increasing protein
CAAD: Carotid artery disease
CAD: Coronary artery disease
CETP: Cholesterol ester transfer protein
CVD: Cardiovascular disease
DNA: Deoxyribonucleic acid
GWAS: Genome-wide association study
HDL-C: High-density lipoprotein cholesterol
LBP: Lipopolysaccharide-binding protein
LDL-C: Low-density lipoprotein cholesterol
LPS: Lipopolysaccharide
PCR: Polymerase chain reaction
PLTP: Phospholipid transfer protein
PLTPa: PLTP activity
RFLP: Restriction fragment length polymorphism
SNP: Single-nucleotide polymorphism
TC: Total cholesterol
TG: Triglyceride
UTR: Untranslated region.
